# Potential Contributions of Psychological Capital to the Research Field of Marketing

**DOI:** 10.3389/fpsyg.2019.02111

**Published:** 2019-09-18

**Authors:** Yu-Lun Lee, Dong-Jenn Yang

**Affiliations:** ^1^Postgraduate Program in Management, I-Shou University, Kaohsiung, Taiwan; ^2^Department of Business Administration, Cheng Shiu University, Kaohsiung, Taiwan

**Keywords:** psychological capital, marketing, sustainability, innovative research, cross-disciplinary studies

## Introduction

Psychological Capital is increasingly becoming an important and integral aspect in the management of most firms across the world with the advent of globalization. There are many components that come within the construct of psychological capital as a whole (Luthans et al., 2007). The interaction of these components yields high levels of efficacy along with motivation of the employees in the organization. These components include hope, self-efficacy, resilience and optimism. These four pillars of psychological capital play different roles in increasing the level of positivity and hence, help in increasing the levels of productivity within different institutions (Avey et al., 2009). These components of psychological capital specifically aim at increasing the level of goal-oriented initiatives taken up by the employees and also help the entire organizational structure to come up with a framework that will help in achieving the goals facing the firm within the stipulated period of time. This paper intends to study the role of these different components and psychological capital as a whole based on the marketing strategies taken up by firms and the related success of such strategies and techniques. Further, the paper is also aimed at understanding the role played by the relationship between psychological capital and marketing in the field of entrepreneurship. The role of psychological capital in the globalization decision of the firms through marketing is analyzed and discussed to understand how such components of decision making can help the new entrepreneurs to succeed faster and reach objectives facing the firm within a shorter period of time.

## Psychological Capital and Marketing

The context of psychological capital is being applied in various contexts within the realm of management operations in order to increase the reach of the business and market the products of the different firms and institutions in ways that will increase the cohort of people belonging to the target market of the firm and hence generate higher revenues along with building a very high brand image. Psychological capital on the whole helps in building a very strong organizational culture (Avey et al., 2008). This section of the paper helps in understanding the contribution of psychological capital to various aspects of a business. It helps in understanding the role of positive psychology in both running and expanding a business (Avey et al., 2006). The level of marketing carried out by the employees of a firm, to a large extent, depends upon the level of job satisfaction that is offered to the marketing managers and other employees related to the marketing of the firm (Peterson et al., 2011). Hence, it is very important to understand the various ways in which the marketing employees of the firm need to be psychologically motivated in order to reach the related objectives. The major focus of this study is on the marketers (i.e., those who's daily life or profession involves deeply with marketing tasks) and the marketing operations of the firm because the level of psychological capital required for employees associated with this type of management is greater as compared to the other job roles because the level of external interaction is larger for such employees (Lee et al., 2017). The employees working in other departments within a corporate institution like finances and engineering do not need to interact and make use of external dynamics in their everyday life.

Below, we selectively discussed some potential intersections of PsyCap and Marketing. Note that these issues are emerged based on our constant and long-run survey of the PsyCap theory and the Marketing field, as well as academic communications with other colleague scholars. Of course, subjectivity should be cautioned as we did not conduct a thorough review of any intersections between PsyCap and Marketing.

### Psychological Capital and Customer Service Quality

Some papers portray the relevance of the concept of positive psychology in developing fruitful long run relationships with the customer base of the firm. There are several means by which customers are able to connect with the firm and become consumers of these products in the long run. This is observed in the case of firms that offer benefits to the society or community in which they operate through corporate social responsibilities. It helps the consumers to psychologically connect to the firm through cognitive, emotional and physical perspectives of benefits (Newman et al., 2014). Such benefit related perspectives help in yielding external motivational factors in the consumers of the firm. There are various theories which help in understanding the concept of engagement of customers with the employees of the firm. One of the most important theories is Kahn's engagement theory. This theory sheds light on the capabilities of employees to harness complete capacity utilization through the psychological conditions of meaningfulness, safety and availability (Blau et al., 2004; Gooty et al., 2009; Liu et al., 2019). This theory asserts that the level of engagement of an employee is more important compared to his everyday hard work. The greater the level of loyalty and feeling of satisfaction derived on psychological levels, the greater is the level of engagement of the employee. This theory also focusses on the fact that the level of engagement keeps changing along with the change in the circumstances in which the employees engage and function. Most studies that deal with psychological capital in terms of enhanced customers service quality assert that the level of customer engagement increases along with an increase in the level of satisfaction amongst the employees as in that case employees are able to deliver better services (Liu et al., 2019).

When the employees are well-engaged with the firm, they are able to play more important roles in the level of customer engagement that the consumers are able to build with the brand. This is so because of the bettering of customer interaction with the brand, the firm and the other customers of the firm. This helps not only in maintaining strong client relations but also in securing new clients which in turn helps firms to increase the size of the target market.

### Psychological Capital and Relation of Employees With the Firm

Most of the studies that deal with the relationship between the variables of employee satisfaction and positive psychological environment tend to reveal that the concept of servant leadership helps in increasing the level of psychological capital within the sales and marketing employees. With practice of this form of leadership, the employees are able to increase their own levels of self-motivation and reduce lateness attitude (Blau et al., 2004). As a matter of fact, this form of leadership helps in developing the psychological needs within employees which causes them to contribute more toward providing better customer service quality and greater marketing of the goods of the firm (Liu et al., 2019). This is in relation to identifying themselves as a part of the firm in the long run for a longer span of time. It is reported that especially in certain industries where the relation between the clients and the sales employees happen on a regular basis, the concept of servant leadership provides huge psychological capital to these employees who interact on almost an everyday basis (Bouzari and Karatepe, 2017). This form of psychological boost is also reported to increase the level of competitiveness of the firm with the help of citizenship behaviors that are completely service oriented. As a matter of fact, it is asserted by other studies that even though the general perception is that in case of management roles that are generally of a higher order and interaction with customers at that level does not need the implementation of psychological capital. However, the presence of importance, respect and accountability plays a large role in bolstering the interaction between such managers and customers of the firm (Newman et al., 2010).

Other studies suggest that it is very important for firms to maintain high levels of resources in order to retain employees that use a high quotient of psychological capital to interact with the customers and hence, improve the existing level of marketing within the firm (Norman et al., 2010). The manner in which human resources are treated and managed also plays a crucial role in the marketing of the firm as a whole in the community in which it operates. The concepts of inclusion, work- life balance, and other concepts that helps in improving the performance and productivity of the employees. The psychological capital quotient of the firm rapidly increases with increase in the number of initiatives taken up by the leaders of the firm for taking care of the concerns and the needs of the employees. According to reports, in the absence of such management systems and policies in place, the employees generally lose the level of commitment and psychological capital which induces negativity and lateness attitude. If this behavior related to tardiness persists in the long run, the average businesses in the United States go through a loss of around $3 billion on an annual basis (Bouzari and Karatepe, 2017). Thus, the variables of psychological capital and job performance even though not vividly explored in the past are related in a way that affects the sustainability and functioning of the firm in the long run. The concepts of job attractiveness, job effort and a balanced corporate life play the roles of catalysts and mediators in increasing the level of psychological capital in the firm.

The role played by psychological capital is more in the case of marketers as they have to deal with a series of challenges and adversities in the external environment of the firm. We agreed that, of course, many other professions have to deal with external challenges and could benefit from owning high-leveled PsyCap. We do not assume that PsyCap is more important to marketers than to other professionals. But we do think PsyCap is of special importance to marketers. The rationale follows. To become a well-equipped marketer, one needs to have the qualities of understanding and empathizing with the consumers which requires high quotients of psychological capital (Sweetman and Luthans, 2010). It is also important for these employees to channelize and use these resources depending on the situation that they are in. The correct set of actions need to be taken up through instant decision making for marketers in order to achieve the resource use for goals facing the firm (Luthans et al., 2008). The same is also true in the exact opposite situations of extreme positive responses from the consumers wherein the marketers have to again channelize the different components of psychological capabilities to modify the situation in order to maintain a balance. The impact of the usage of skills pertaining to psychological capital has been found to be maximum in case of transition economies because of a number of underlying reasons including cultural shift and demographic shifts that such economies go through (Mathe et al., 2017). The impact of psychological capital on the job performance also varies depending upon the sector in which the firm is established and is operating. Most studies assert that hiring the right kind of marketers is important and should not only depend upon their educational levels and specialized skills attained but also on their level of psychological capital (Sulistyo, 2016). In addition to this, fostering and nurturing a high quotient of psychological capital is also important for firms and this should be managed and promoted with the help of sessions, conferences and trainings provided specifically to employees working or associated with the marketing departments of firms. To harness an environment that protects and promotes high levels of psychological capital, it is important to formulate and maintain appropriate policies and practices for efficient human resources management. Thus, human resources such as the marketers play a crucial indirectly in the marketing of products and dealing with clients of the firm.

Some of the business functions tend to focus on the allocation of the sales employees with different customers for different products based on the relatedness of the employees to the products, the experience level and the level of competence that the employee is associated with. Proper allocation based on the psychological capital helps these employees to exercise different levels of freedom and make use of their opinions which is reported to be successful in most cases (Paek et al., 2015). This has been proven to be especially true for the workers who have been able to build cordial and strong relations with the higher authorities of the firm including the management. It is reported that they possess greater amounts of psychological capital and are able to make the most out of challenging situations. These employees are able to figure out methods and strategies in a very small span of time if the initial strategies developed by them fail to reach the desired outcomes. The probability of these employees to bounce back from adverse situations is reported to be higher than most other employees. This forms a very important strength in the competitive advantage context for the firm.

Moreover, psychological capital is reported to be one of the best and most efficient predictors of performance of the employees employed especially in the marketing department (Paek et al., 2015; Bouzari and Karatepe, 2017). Generally, firms are concerned with using the values of psychological capital for predicting the nature of productivity of the employees and not the four individual components that form the psychological capital.

### Psychological Capital and Innovation

Innovation such as new product development has been a critical part of marketing research. It has been found from most studies that the capacity of innovation is not enough for a firm to be able to successfully able to grow and develop (Chen and Lim, 2012). Simultaneously, efficient marketing skills are also required for gaining market exposure and brand value. This, in turn, requires the implementation of strategic and well-decided methods of marketing using psychological capital. Studies assert that in some specific countries like Canada, innovation requires a lot of investment in the marketing and advertising of goods and not only technological improvisation (Sweetman et al., 2011). Marketing in this context refers to a gamut of events including building up connections to increase the reach of the product and building a strong promotional network (Cole et al., 2009). Studies have asserted that the usage of positive psychology capital for innovation in the field of marketing is extremely important for achieving greater levels of competitive advantage and firm performance (Mathe et al., 2017).

Psychological capital in the context of marketing innovation involves the participation of various components of customer engagement (Nguyen and Nguyen, 2012). It involves consumers, suppliers, trademarks, and industry associations. Ensuring that all of these components are working together in an efficient and effective manner involves the implementation of psychological capital in the form of social capital. As a matter of fact, studies conducted in Italy portray that the production processes of firms are handled in a systematic manner with the application of psychological capital (Na Ayutthaya et al., 2016). This concept is a very useful tool for the transitioning economies as continuous innovation has to occur in these firms depending upon the continuously changing demands and preferences on the part of the consumers. Most of the knowledge required for innovation in terms of marketing comes from the usage of tools and techniques that render knowledge about the common strategies taken up by the majority of firms in the same industry with the help of pooling of psychological or social capital.

In countries like Malaysia, for example, it is generally noticed that firms need to bring together and equate the variables of social capital and mission culture in order to achieve certain goals in terms of growth facing the organization in the long run (Lifeng, 2007; Hur et al., 2016). The amount of resilience also increases with the application of proper psychological capital which is equally important as diversified adversities are faced in the process of both technological and marketing innovation. With the help of the right strategies, the employees are able to connect to the customers and solve their problems in different ways at different points of time, which in turn allow them to retain themselves as well as the customers in the long run. As a matter of fact, it is observed that the level of psychological capital also helps in framing the type of empathetic response that most marketers need to use for solving different customer problems and challenges posed by the competitors. This helps a lot with the process of innovation as a lot of data can be used and the challenges persisting in the industry can be overcome.

## Propositions for the Future Research Directions

Although the concept of using psychological capital is becoming increasingly common with the advent of development and globalization all over the world, it is still comparatively a new concept that provokes interest and there are many aspects of using psychological capital in relation to the field of marketing that need to be addressed and future research should be taken up in those areas. A few such propositions aiming at future research based on these concepts are discussed in this section of the paper which helps in shedding light on the areas yet not researched.

Firstly, the most common and important method of research that can be undertaken involves the analysis of data related to relation between the variables of psychological capital and marketing objectives with the usage of time series data or even panel data over a substantially long period of time as compared to the current situation. This is crucial as this dynamic equation between the two variables is comparatively new and needs to be explored across different regions of the world over longer periods of time as these equations have been newly discovered and are being worked upon in the current span of time.

Secondly, studies should also be taken up in the form of comparison between the impact psychological capital has on marketing directly and the impact it has on employees that are associated with marketing for a firm in an indirect manner. In other words, research based on the differences in the impact of psychological capital on the back house and front house operations in the marketing department of different industries where both the operational systems play a vital role, like the hospitality industry should also be taken up. This will help in understanding the importance of positive psychological capital from different perspectives.

Research can also be taken up based on the role played by psychological capital in terms of the relation between psychological capital's impact on other business functions like accounting and finance. This should be further be compared to the correlation between marketing and psychological capital such that the role played by psychological capital on these different business functions. These dynamics can further be used to enhance the strategies taken up to reach goals facing the firm using proper allocation of psychological capital in the different departments within the same firm.

There are other areas of research pertaining to psychological capital quotient of the marketers. These include components of personality apart from the four components of hope, self-efficacy, resilience and optimism. Research should be taken up in the future pertaining to these personality traits including their individual personalities and hardiness in terms of their individual psychological constructs. The role played by such traits in shaping or enhancing the characteristics of psychological capital of the marketers should also be taken up. Such research will also bring out the factors that motivate the marketers in terms of new challenges and work environment offered to them.

Studies related to the understanding the innovative capability of the employees especially in creative industries in relation to the level of psychological capital that they possess should also be taken up. This can further be correlated with the competitive advantage of the firm to investigate and analyse the performance of the firm in the long run. The success of the firm related to the innovative capabilities along with the role played by psychological capital can help find important insights in terms of hiring the right fit of employees for the long run success of the firm.

The level of engagement and involvement of employees within the various functions and operations of different firms and the satisfaction received by the same employees are considered to be important constructs in terms of psychological capital that motivates employees toward institutional commitment. A mechanism that helps in studying the structural relationships in between such constructs can also be studied in future researches. This will help in understanding the role of the human resources management in shaping the psychological constructs of the organization under consideration.

Moreover, there are many studies that directly relate the variables of innovation and competitive advantage to find out the rate of success of the firm. Future research can also involve investigating the relationship between social capital and long run success of the firm with usage of innovation capacity being treated as an intervening variable. This process of research can be implemented in various industries to find out the industries where the correlation between these three variables is the highest.

Studies can also be taken up in terms of the role played by psychological capital in shaping the level of customer engagement in different industries over a longer span of time. Understanding how the level of customer engagement varies over time for a particular firm and comparing this with the results from another firm in another industry will help in understanding the differences in ways which should be used for allocating and implementing psychological capital over time for marketing products and increasing the level of customer engagement.

One of the most important fields of research can be in the field of finance marketing. As most of the research has been conducted to find out the relation between psychological capital and customer engagement and marketing for firms, further research should involve relating the success in terms of brand development and marketing of products to the financial products/services success of the firm in both the short run and the long run. These researches can be used for portraying the relationship between the customer relations developed through the four components of psychological capital and the financial attributes that determine the overall success of the firm. Moreover, most of the data used is from the perspective of firms. More studies should be conducted using the perspective of consumers as to how the behavior and the marketing techniques help them to choose products or firms better, especially in the long run.

Studies can also be taken up to explore further conditions and variables within a firm that affect the development and implementation of psychological capital within the employees. These can include the environment of the organizational culture and the type of hierarchical structure followed in the firms under study. Studies can also be undertaken to understand the impact of other business employees operating and functioning in other departments on the building of psychological capital in the employees operating in the marketing sector of the firm.

Another field of research where extensive development can be done involves the study of the role of psychological capital with marketing across employees belonging to different age cohorts and across different geographical areas of the world.

## Extension: From Sustainable Marketing to Entrepreneurship

With increased levels of technological innovation and increased barriers to entry for new and budding entrepreneurs, the level of research being conducted in the paradigm of psychological capital and entrepreneurship though less is gradually gaining momentum. Psychological capital is considered to be more related to the context of human resources and job satisfaction (Clapp-Smith et al., 2009). However, business and entrepreneurship are conducted in a process-oriented manner and depend on a set of factors that does not involve the four major components of psychological capital. Instead, these factors include factors like availability of opportunities, development and utilization of available opportunities (Woolley et al., 2011). It is often observed that factors like entrepreneurial ability and venture performance are given more importance unlike the concept of psychological capital. It is used as an explanatory variable in few researches and the idea of relating the success of individual business with psychological capital is increasingly becoming important and necessary (Abbas et al., 2014). However, the history of relating psychological capital with the performance of joint ventures has been traditional and is yet being explored. Studies conducted on these lines have claimed that as compared to human and social capital, entrepreneurial capital changes the way the venture performs and causes wide variations (Walumbwa et al., 2010). This is caused due to the dynamic external environment in which the entrepreneur performs his business operations. It is asserted in such studies that the higher the level of dynamism in the external environment of the business, the higher is the correlation in between the variables of psychological capital and venture performance.

There are studies that help in understanding the simplest form of relation between the constructs of psychological capital and individual entrepreneurship. It is widely observed that most new and budding entrepreneurs fail to make the business establish and are often forced to exit the market in which they operate (Ming and Zuguang, 2013). This is because of the lack of proper forecasts and long run sustainable business operations. However, in spite of such problems, the firms and the entrepreneurs are individually still choosing to take up risks and set up their individual ventures in the face of such risks and challenges. This is because of the capacity of these entrepreneurs to make use of the psychological capital they possess in terms of self-confidence and self-efficacy (Roche et al., 2014). This is the basis for the setting up of numerous businesses and expansion of already established businesses all over the world.

In terms of the resource endowments of the entrepreneurs, the level of human resources possessed plays a crucial role in the formation of a well-established business in the long run. With the implementation of proper strategies in terms of human resources along with business development strategies, the businesses are noticed to survive in the long run. This is because of the crucial role played by psychological capital on the part of both the employees and the managers related to the business as well as the customers that are trying to build loyalty toward the new products and services of the firm (Kim et al., 2017). Moreover, the success of a potential business involves the use of effective leadership policies which are authentic and help in understanding the social cognitive framework in an easier manner with the help of psychological capital itself (Kobayashi et al., 2006). These leadership policies are mostly focused on the usage of proper human and psychological capital such that their deliberate usage is known to increase the level of growth for the firm in the initial years of setting up the business. As is observed in the earlier sections of the research, strategic use of psychological capital and other psychological resources help in attaining greater levels of customer engagement which is important for the firm in the initial years of setting up the business and even developing the brand value.

Moreover, psychological capital is also known to help in dealing better with the internal challenges of the firm for the entrepreneurs that saves a lot of time and resources for proper implementation in other areas of establishing the business. The use of goal directed mechanisms involve implementation of positive psychology in the initial years of business. As a matter of fact, after a few years, psychological capital can be used as a process of self-evaluation for the process of business (Dinh Tho et al., 2014). The use of psychological capital has in fact been found to affect the entrepreneurial decisions in a positive manner. The level of optimism also helps to go through the various challenges faced in the beginning of setting up the business. Proper usage of psychological capital helps in increasing the level of curiosity which itself helps the firm to overcome the problems of failure and exit from the market. It helps the entrepreneurs to build better disruptive business models which can be used for collection and analysis of data which in turn helps to create solutions to most of the problems perceived on entry into the market. The use of psychological capital is also reported to better the level of organizational orientation and organizational performance.

The different components of psychological capital help to make the locus of control of the business stronger and more solution oriented. It also helps in creating a more conducive environment for the employees so that they are able to make full capacity utilization and increase the level of productivity (Karatepe and Karadas, 2015). One of the most important parts of setting up a business involves the setting up of a set of values whose integral significance remains the same over a particular span of time and which are also flexible in the long run such that with changes being introduced in the system, these values can also change. The component of hope in psychological capital helps in framing such values with the help of strategies that help in attaining and achieving goals lying ahead of the business operations in the upcoming years.

Some other studies assert that the role of both psychological and start-up capital is important in going on with a business as both help in predicting the achievable level of success in the first few years of establishing the business (Jung and Yoon, 2015). The component of psychological capital that helps in bringing together the components of startup capital and customer engagement is that of optimism. It helps in connecting the business to the needs of the customers which in turn helps the firm to provide efficient and effective services to the consumers and increase the standard of living of the community as a whole in which the firm operates. Psychological strength is extremely important for even setting up the infrastructure and arranging other start up contacts and sponsors in case of small businesses. Studies also reveal that psychological capital plays an important role in taking care of the people involved in setting businesses on an individual basis (Perkins et al., 2002). It helps different people in different ways. However, at the aggregate level, it helps to connect to the customers at an empathetic level and gain increased reach in the beginning years which is extremely important for building a base of loyal customers as well as a high brand value.

The level of psychological capital affecting the setting up and running of a particular business also depends on a lot of other conditions including the industry in which the firm operates, the geographical reach of the firm and even the culture of work that is to be followed while setting up the business (Jancenelle et al., 2018). Even to a larger context, psychological capital could influence marketing phenomena. For example, Chen and Wu (Chen and Wu, 2019) proposed that psychological capital is a foundation of food safety's social co-governance mechanisms, which incorporated and coordinate among multiple stakeholders in the society (e.g., government, business, consumers, third-parties, etc. It could also be reflected by studies that as the level of psychological capital varies across different countries and different types of employees, the level to which it is used for the establishment and functioning of different businesses also differs. As a matter of fact, the age to which the different employees of a firm belong also plays a vital role in the relation between the psychological capital and the success of the firm in the long run as positive psychology plays completely role across different ages of the human demography.

## Conclusion

In order to conclude, it can be asserted that psychological capital is increasingly playing a very important role in the field of both management and entrepreneurship. It helps in connecting a potential business to its customers and also helps the consumers to understand the functioning of a business better. It has been found that most firms are not able to connect or use the human resources or other resources available to them using psychological capital. However, this variable is reported to help in a lot of operations including motivating employees and increasing productivity for the firms. It also helps the firms to increase the level of customer engagement while bettering the level of customer service quality. Reports suggest that most firms that implement the usage of psychological capital are also able to innovate more, not only in terms of technological improvisation but also in terms of marketing of the products and services created by them. As a matter of fact, the firms are trying to increasingly use the values of psychological capital as an intervening variable to relate the level of innovation to the level of customer engagement through efficient marketing. Although this is a new and increasingly interesting area of research, more work based in the field of research needs to be taken up using various nuances of business that relate psychological capital to efficient marketing. Studies also need to be taken up in order to understand the relations between the impact of psychological capital in between marketing and the various other related business functions. As the study is increasingly becoming important, future research pertaining to the analysis of long run data will also help in finding out trends that shape the relation between marketing and psychological capital and how the contribution to the realm of marketing changes over time.

## Author Contributions

All authors listed have made a substantial, direct and intellectual contribution to the work, and approved it for publication.

### Conflict of Interest Statement

The authors declare that the research was conducted in the absence of any commercial or financial relationships that could be construed as a potential conflict of interest.
